# The Corona crisis: a wicked problem

**DOI:** 10.1177/1403494820970767

**Published:** 2020-11-17

**Authors:** Per Morten Schiefloe

**Affiliations:** NTNU Social Research, Norwegian University of Science and Technology, Trondheim, Norway

**Keywords:** corona crisis, wicked problem, complexity theory

## Abstract

The aim of this paper is to analyse the current Corona crisis from the perspective of the theory of wicked problems. The analysis is based on a combination of observation of national and global effects of the pandemic and a study of relevant theoretical contributions. The findings confirm that the crisis is of a kind that corresponds to the main characteristics of the wicked problems theory. The conclusion is that the pandemic cannot be approached by standardised analytical techniques, because it, like other wicked problems, represents a unique challenge and because all possible solutions may lead to unknown negative consequences.

Sometimes politicians – and society at large – face a problem without precedent, and where there are no proved recipes that can guarantee a good solution, because the problem is ‘wicked’. Wicked must here be understood as resistance to resolution, rather than as evil.

The concept of wicked problems was introduced in 1973 by Berkeley professors Rittel and Webber in a paper addressing dilemmas in planning and social policy [1]. Since then, the concept has become widely used, especially in policy analysis, but also in research on safety and vulnerability [2–4].

Rittel and Webber’s main argument was that many of the emerging societal problems that confront contemporary planners and policymakers are of a kind that defy the capacity of governments to find good solutions through the kind of processes that are typically used. These emerging problems do not fit into established functional sectors, such as healthcare or education. Neither can they be defined in terms of instruments that may be used to address them – such as new formal regulations or increased funding [5]. In addition, they are often contested and debated, and are characterised by disagreements both on the definition of problems and on solutions [6].

A problem becomes wicked because of the incomplete knowledge of effects and interdependencies, because it involves actors operating in different sectors and at different levels, because all possible actions have uncertain effects, and because they are intertwined with other problems in complex and, to a large extent, unmanageable systems. Such problems are different from hard but ordinary problems, which can be approached and solved by standard techniques. Conventional problem-solving processes, however, do not only fail to tackle wicked problems. They may also make things worse by generating undesirable consequences [7]. Illustrating examples are environmental degradation, Islamic terrorism and illegal immigration. And today: the Corona crisis.

In their original paper Rittel and Webber identified 10 primary characteristics of wicked problems. Five of these characteristics are of special relevance here:

1. There is no definitive formulation of a wicked problem. This means that the problem cannot be isolated and approached by standard analytical methods. Even if the initiating factor for the current crisis – a new virus – is known, the consequences are almost indefinite, reaching into almost all sectors of society; the health system, education, trade, service, tourism and industry. The crisis is also deeply affecting our private and social lives, and not only through illness and death.
2. Every wicked problem is essentially unique and represents unknown challenges. There may have been previous problems of a similar kind, but despite possible similarities there are overriding differences. This is obviously the case in the present situation. Pandemics are a well-known phenomenon. The ‘Spanish flu’ is a prominent example. What is different this time is the rapid spread of the virus in a tightly coupled global economy where ‘everything is dependent on everything’. The effect on the globalised production chains for medicine and protective equipment illustrates this clearly.
3. Because the problem is new and has other effects than what has been experienced earlier, there are no proved solutions. Solutions can neither be found through established, rational decision-making procedures. This is clearly illustrated by the fact that different medical experts come up with different propositions and that medical arguments must be weighed up against economic and political considerations. One effect of this is that different countries and regions come up with different approaches.
4. From this follows that there are no true or false decisions, decisions are only better or worse. Decisions that may be ‘right’ seen from a perspective of infection control may be devastating to the economy and unacceptable from a political viewpoint. And the other way around. Neither can possible solutions be tried out through experiments or on a small scale. There is no opportunity to learn by trial and error. Not before this is all over is it possible to decide what national strategies have led to the best overall outcomes.
5. All attempts at solutions may have incalculable and irreversible consequences. When large parts of the economic activities are shut down, we don’t know what effects this will have, neither in the short or in the long run. Maybe some kinds of business will never return to what they were before. This is the ‘Catch 22’ syndrome in wicked problems [8]: In order to find solutions, they must be tried out, but every attempt is expensive and may lead to unknown negative consequences, which again may cause new wicked problems. An illustration in this case is the closing of schools and kindergartens combined with compulsory quarantine for whole families and the possible negative effects, both health-related and social, that this can have for vulnerable children. Other negative effects are increased levels of domestic violence and mental effects of unemployment and economic stress.

Wicked problems are inherently different from problems in fields such as engineering, natural sciences and medicine, where solutions usually can be developed by means of well-known techniques for analysis and decision-making. Rittel and Webber characterise problems in which such standardised problem-solving can be applied as ‘tame’ [1]. A tame problem can be difficult, but it is well defined, has a solution which can be objectively evaluated as right or wrong and has a definite stopping point when the solution is reached. A tame problem belongs to a class of problems which may be solved in similar ways and comes with a limited set of alternative solutions which can be tried and abandoned if they don’t work. A typical illustration for handling such problems is the ‘waterfall model’ which is used in developing software [8]. This model depicts the progress from problem to solution as flowing down several steps, following a logical order; specifying the problem, gathering data, analysis, formulating alternative solutions, decision-making and implementation. The analytical groundwork is usually taken care of by expert agencies in the public administration or external consultants, whereas politicians make the final decisions. Such processes are often also legally regulated. These are the kinds of problems that the political and administrative systems are used to handle. Alternative solutions to tame problems can also be evaluated based on different ideological viewpoints.

The recognition of wicked problems can to some extent be seen as related to the development of complexity theories in the social sciences [8]. Like in complexity theory, the original description by Rittel and Webber focuses on systems in which the relationships between variables are not linear and where small disturbances and shifts in the initial phases of incidents may have large and incalculable consequences. In popular language this is often called ‘the butterfly effect’ [9]. In this case the butterfly was probably the transfer of a virus from a bat to another animal and then to a human in a Chinese food market.

Another important point is that complex societal systems tend to involve multiple actors with different roles and interests, and therefore can be politically complex [10]. An example is how the polarisation in American politics strongly influences the attitudes to the danger posed by the virus, both within the political system and among ordinary citizens [11].The attempts by local Chinese authorities to silence the first whistle-blowers and hide the outbreak of the pandemic is also a clear illustration.

The description of wicked problems and dynamic systems bears some resemblance to Perrow’s theory on ‘normal accidents’ [12]. Perrow’s main argument is that when sociotechnical systems are complex, tightly coupled and have catastrophic potential, accidents are to a large extent inevitable outcomes. Characteristic of a complex and tightly coupled system is the absence of ‘natural buffers’ and that there are limited opportunities for containing initial disturbances through improvisation. This implies that small beginnings can turn into catastrophes through cascading effects. Perrow also argues that such systems display an inevitable dilemma in the balance between centralised and decentralised authority: The fact is that a system with high interactive complexity can only be effectively controlled by a decentralised organisation, whereas a system with tight couplings can only be effectively controlled by a highly centralised organisation. Perrow’s theory is based on studies of complex industrial organisations, such as nuclear power plants, but his basic concepts and reasoning are clearly applicable also for understanding important aspects of the present situation.

In a paper where they analyse the political inability to cope with carbon dioxide emissions and global warming, Levin et al. introduce the concept of ‘super wicked problems’ to characterise a new class of global environmental problems [13]. In addition to the attributes of wicked problems described by Rittel and Webber [1] they point to four key features, here slightly reformulated to fit the present situation:

1. We are in a hurry – time is running out, and there is no time to wait for ordinary political processes with different stakeholders, conflicting interests and different constructions of reality. The time dimension means that the problem can spin out of control, have too much impact and be followed by consequences that will be impossible to reverse.
2. Those who must act to end the problem are also causing it. The pandemic can only be controlled by individual behaviour, but at the same time it is individual behaviour that causes the spread of the virus. Government can try to restrain and influence individual behaviour, but never control it completely. This is clearly illustrated in the rise in new infections in Europe following summer holidays and by increasing popular protests against lockdowns and other Corona restrictions.
3. There is no effective coupling between system levels causing or affecting the problem and the system levels to control them. While the pandemic is a challenge on the global level, it is up to national and regional authorities to act. There is no central authority.
4. Policies discount the future irrationally. The political system operates within short time spans and seldom looks beyond the next election. Even in the face of clear evidence of risks and the probability of catastrophic impacts from inaction, political decisions tend to reflect limited time horizons and immediate and visible gains.

Pandemics are not unknown events and the Corona virus is no Black Swan. An illustration is that the Norwegian Directorate for Civil Protection ranks the risk for a pandemic on top of their list of possible crises that can threaten the Norwegian society [14]. This ranking is based on a standard two-dimensional risk matrix combining probabilities and consequences. Pandemic is ranked with the highest value (5) on consequences and the second highest (4) on probability. In second place, with value (4) on both dimensions, they place shortage of medicine. Based on experiences from the recent months we can here also add shortages of medical equipment. The challenge for the political system is that investments in emergency preparedness compete with all kinds of ‘good and popular purposes’ and that investments in societal insurance, such as extra stocks of protective equipment and respirators, remain invisible for the electorate if nothing happens. As stated in a recent conversation with the CEO of a large public organisation – ‘you don’t get re-elected because the roof is not leaking’.

Contrary to negative stereotypes, government organisations often function well when it comes to implementing policies and delivering services that are relatively standardised, such as infrastructures, health and education. They are, however, usually less well equipped when they are faced with new and non-routine challenges. And this is especially true when the challenges meet the criteria of being wicked [15].

There is no proved recipe for handling wicked problems, but collaboration and coordination between different actors, organisations and administrative levels are often seen as preconditions for being able to address such complex governance challenges [6]. A relevant term is ‘collaborative advantage’ [16]. If collaboration between all involved parties is operating effectively, it can help in addressing wicked problems in three ways. First, functioning cooperative networks increase the likelihood that the nature of the problem can be better understood. Second, collaboration increases the possibility that provisional solutions can be found and agreed upon. Third, good collaboration facilitates the implementation of solutions, both because the different actors have agreed on what to do, but also by facilitating coordinated actions and mutual adjustments [15].

As the concept of wicked problems has attracted increasing attention, it has become widely used in policy analysis. Peters and Tarpey argue that the popularity of the concept has led to an overuse, which they characterise as ‘conceptual stretching’. Based on a survey among policy experts, they find that there are few, if any, policy problems that have all the attributes originally formulated by Rittel and Webber [5]. This seems, however, definitely to be the case when the political and administrative systems are confronted with the Corona pandemic. The pandemic is not only a wicked problem, it is also qualifying as ‘super wicked’.
